# Role of leukocytes and systemic inflammation indexes (NLR, PLR, MLP, dNLR, NLPR, AISI, SIR-I, and SII) on admission predicts in-hospital mortality in non-elderly and elderly COVID-19 patients

**DOI:** 10.3389/fmed.2022.916453

**Published:** 2022-08-18

**Authors:** Hassan Ghobadi, Jafar Mohammadshahi, Nazli Javaheri, Nasrin Fouladi, Yasaman Mirzazadeh, Mohammad Reza Aslani

**Affiliations:** ^1^Lung Diseases Research Center, Ardabil University of Medical Sciences, Ardabil, Iran; ^2^Department of Infectious Diseases and Tropical Medicine, School of Medicine, Ardabil University of Medical Sciences, Ardabil, Iran; ^3^Department of Internal Medicine, School of Medicine, Ardabil University of Medical Sciences, Ardabil, Iran; ^4^School of Medicine, Social Determinants of Health Research Center, Ardabil University of Medical Sciences, Ardabil, Iran; ^5^Faculty of Medicine, Ardabil University of Medical Sciences, Ardabil, Iran; ^6^Applied Biomedical Research Center, Mashhad University of Medical Sciences, Mashhad, Iran

**Keywords:** coronavirus, COVID-19, inflammation, aging, systemic inflammation index

## Abstract

**Background:**

Systemic inflammation indices, including neutrophil/lymphocyte ratio (NLR), monocyte/lymphocyte ratio (MLR), platelet/lymphocyte ratio (PLR), derived neutrophil/lymphocyte ratio (dNLR), neutrophil/lymphocyte^*^platelet ratio (NLPR), aggregate index of systemic inflammation (AISI), systemic inflammation response index (SIR-I), and systemic inflammation index (SII) are well-expressed inflammatory indices that have been used to predict the severity and mortality of various inflammatory diseases. This study aimed to investigate the role of systemic inflammatory markers in predicting mortality in non-elderly and elderly COVID-19 patients.

**Methods:**

In a retrospective study, laboratory parameters were examined for 1,792 COVID-19 patients (elderly = 710 and non-elderly = 1,082). The ability of inflammatory markers to distinguish the severity of COVID-19 was determined by receiver operating characteristic (ROC) analysis, and survival probability was determined by the mean of Kaplan–Meier curves, with the endpoint being death.

**Results:**

In the non-survivor non-elderly and elderly patients, the parameters PLR, MLR, dNLR, NLPR, AISI, SIR-I, and SII were significantly higher than in the surviving patients. WBC count (HR = 4.668, 95% CI = 1.624 to 13.413, *P* < 0.01), neutrophil count (HR = 6.395, 95% CI = 2.070 to 19.760, *P* < 0.01), dNLR (HR = 0.390, 95% CI = 0.182 to 0.835, *P* < 0.05), and SII (HR = 10.725, 95% CI = 1.076 to 106.826, *P* < 0.05) were significantly associated with survival. On the other hand, in elderly patients, it was found that WBC count (HR = 4.076, 95% CI = 2.176 to 7.637, *P* < 0.001) and neutrophil count (HR = 2.412, 95% CI = 1.252 to 4.647, *P* < 0.01) were significantly associated with survival.

**Conclusion:**

WBC count and neutrophil count in non-elderly and elderly patients, were reliable predictors of mortality.

## Introduction

Symptoms of coronavirus disease 2019 (COVID-19) in patients are reported to vary from minor symptoms (such as headache, fatigue, and fever) to severe symptoms (such as dyspnea or hypoxia) (1–4). With this wide range of clinical characteristics and disease outcomes, differences between non-elderly and old patients have also been reported (1, 5–7). Various studies have shown that old age is associated with high disease severity and mortality (8). Due to physiological and pharmacological changes as well as comorbidities such as diabetes, cardiovascular disease, lung disease, and kidney failure, elderly people have a high risk of severe illness and hospitalization in the intensive care unit (ICU) compared to non-elderly individuals (9, 10). Because the elderly have specific clinical features, such as cognitive and behavioral disorders, chronic underlying diseases, and clinical manifestations with non-specific and unusual symptoms, it is challenging to diagnose COVID-19 in them accurately (11, 12).

Recently, neutrophil/lymphocyte ratio (NLR), monocyte/lymphocyte ratio (MLR), platelet/lymphocyte ratio (PLR), derived neutrophil/lymphocyte ratio (dNLR), neutrophil/lymphocyte^*^platelet ratio (NLPR), and aggregate index of systemic inflammation (AISI) are novel inflammatory markers, which are considered in the diagnosis and progression of a variety of inflammatory and infectious diseases, including COVID-19 infection (13–15). Also, systemic inflammation response index (SIR-I) and systemic inflammation index (SII) are other inflammatory markers that can be used to predict the severity of COVID-19 disease. The predictive value of mortality of these inflammatory markers in COVID-19 old patients compared to non-elderly patients is unclear. Therefore, the present study aimed to evaluate NLR, PLR, MLR, dNLR, NLPR, AISI, SIR-I, and SII on admission in predicting mortality in COVID-19 elderly and non-elderly patients.

## Methods

In a retrospective study, all patients with confirmed COVID-19 in Ardabil Imam Khomeini hospital, northwestern Iran, were included from September to November 2020. The diagnosis of COVID-19 was based on a positive PCR test, and suspected cases were excluded. Patients were divided into two groups: non-elderly (18–65 years) and elderly (≥65 years). The study was conducted after the approval of the ethics committee of Ardabil University of Medical Sciences (IR.ARUMS.REC.1399.615).

### Data collection

Data were obtained from the electronic medical record system of Imam Khomeini Hospital of Ardabil University of Medical Sciences, including sex, age, clinical symptoms, medical history, comorbidities, signs, laboratory tests, duration of hospitalization, and outcome of the disease (recovery or death). To accurately collect patient information, two trained medical students double-checked electronic information. Laboratory tests performed in the first 24 h of hospitalization included complete blood count, coagulation profile, renal function, liver function, and parameters related to inflammation [ferritin, erythrocyte sedimentation rate (ESR), D-dimer, lactate dehydrogenase (LDH), and alkaline phosphatase (ALP)].

NLR, PLR, and MLR, as well as derived NLR (dNLR) [neutrophils/(white blood cells - neutrophils)], NLPR [neutrophil/(lymphocyte ^*^ platelet)], SIR-I [(neutrophils^*^monocytes)/lymphocytes] and SII [(neutrophils ^*^ platelets)/lymphocytes], were calculated for all subjects.

### Data analysis

Data analysis was performed using SPSS software version 21 and MedCalc version 19.4.1. Mean ± standard deviation (SD) were used to present normally distributed variables, and median and interquartile range (IQR) values were used for abnormally distributed variables, while categorical variables were reported as percentages. To compare the continuous variables, independent group *t*-tests (data were normally distributed) and the Mann–Whitney test (data were not normally distributed) were used. Receiver operating characteristics (ROC) curve analysis was performed to estimate optimal cut-off values, maximizing sensitivity and specificity according to the Youden index. For survival analysis, time zero was defined as the time of hospital admission.

Inflammatory indices derived from blood cell counts were also evaluated separately to avoid linear bias by univariate analysis for age, disease severity, and Charlson's index, and confounding factors were corrected if there was *P* < 0.2. Survival probability for CBC-derived inflammation indexes was estimated using the means of the Kaplan–Meier curves, with the endpoint being death. Cox proportional hazards regression was performed for both univariate and multivariate analyses.

## Results

### Demographics characteristics

One thousand seven hundred ninety-two patients admitted with COVID-19 were included in the current study, including 1,082 non-elderly patients and 710 elderly patients. The mean age of non-elderly patients was (48.35 ± 11.61) and old patients were (76.29 ± 6.95).

The percentage of female in COVID-19 non-elderly patients (44.9) was significantly higher than the elderly patients (41.2%, *P* < 0.001). Although elderly patients had a more extended mean hospital stay (8.44 ± 6.81) than non-elderly patients (7.90 ± 7.48), the difference was insignificant. The characteristics and demographics of all patients are summarized in Table 1.

**Table 1:** Demographic, hematological, and blood cell count-derived inflammation indexes of non-elderly and elderly COVID-19.

**Variables**		**COVID-19**		***P*-value**
	**Normal range**	**Non-elderly patients (*n* = 1,082)**	**Elderly patients (*n* = 710)**	
Age	–	48.35 ± 11.61	76.29 ± 6.95	0.000
**Sex**	–			0.000
Male, *N* (%)		636 (58.8)	352 (49.6)	
Female, *N* (%)		446 (41.2)	358 (50.4)	
Hospitalization stay	–	7.90 ± 7.48	8.44 ± 6.81	0.122
WBC (×10^9^/L)	3.5–9.5	7.52 (7.18–7.92)	8.24 (7.88–8.67)	0.000
Neutrophil (×10^9^/L)	1.8–6.3	5.90 (5.10–6.51)	6.68 (5.91–7.30)	0.000
Lymphocyte (×10^9^/L)	1.1–3.2	1.26 (0.80–1.88)	1.11 (0.75–1.71)	0.002
Eosinophil (×10^9^/L)	<0.5	0.13 (0.07–0.16)	0.15 (0.07–0.17)	0.000
Monocyte (×10^9^/L)	0.2–0.3	0.21 (0.14–0.31)	0.24 (0.15–0.33)	0.000
Hb (mg/ml)	11.5–15	13.30 (11.9–14.6)	12.9 (11.4–14.4)	0.001
Hct (%)	36–48	39 (35.8–42.7)	38.8 (34.7–42.6)	0.208
PLT (×10^9^/L)	125–350	192 (147–250)	183 (139–250)	0.120
PT (s)	11–13.5	12.5 (12–13.2)	13 (12–14)	0.000
PTT	30–40	30 (30–36)	30 (30–37.5)	0.294
INR	0.8–1.1	1 (1–1.10)	1 (1–1.20)	0.000
ALT (IU/L)	7–40	38 (25–60)	32 (20–51)	0.000
AST (IU/L)	0–45	48 (33–70)	48 (33.5–77)	0.392
LDH (IU/L)	114–240	659 (498–846)	665 (495–845)	0.716
Ferritin (μg/L)	11–330	630 (283–984)	637 (294–1120)	0.314
ESR (mm/h)	0–29	45 (31–63)	47.5 (32–63.5)	0.221
BG (mg/ml)	70–100	114 (99–152)	133 (105–198)	0.000
Urea (mg/mL)	6–24	31 (24–43)	53 (37–76.5)	0.000
Cr (mg/mL)	0.5–1.2	0.9 (0.80–1.10)	1.2 (0.9–1.70)	0.000
D.Dimer (mg/L)	0–0.5	0.5 (0.2–1.2)	0.59 (0.25–1.06)	0.313
Albumin (g/mL)	4–5.5	3.5 (2.9–3.8)	3.2 (2.6–3.6)	0.001
ALP (IU/L)	44–147	174 (138–233)	193 (152–260)	0.000
Na (mEq/L)	135–145	140 (137–142)	139 (136–142)	0.011
K (mEq/L)	3.5–5.3	4 (3.7–4.2)	4.1 (3.8–4.7)	0.000
NLR		4.7 (2.8–8)	6.07 (3.55–9)	0.000
PLR		154 (92–261)	168 (94–282)	0.118
MLR		0.16 (0.1–0.27)	0.20 (0.11–0.31)	0.000
SIR-I		0.95 (0.52–1.64)	1.28 (0.73–2.19)	0.000
SII		883 (479–1656)	1136 (568–1990)	0.000
dNLR		3.54 (2.33–5.66)	4.26 (2.84–6.69)	0.000
NLPR		0.02 (0.01–0.04)	0.03 (0.01–0.05)	0.000
AISI		183 (84–347)	234 (120–454)	0.000
**Severity**	–			0.000
Moderate, *N* (%)		861 (79.6)	452 (63.7)	
Severe, *N* (%)		86 (7.9)	76 (10.7)	
Very severe, *N* (%)		135 (12.5)	182 (25.6)	
**Comorbidities**				
Cardiovascular disease (%)		11.3	34.2	0.000
Respiratory disease (%)		14.4	15.8	0.235
Kidney disease (%)		5.5	14.2	0.000
Diabetes (%)		20.4	42.4	0.000
Cancer (%)		3.6	4.6	0.164
Charlson comorbidity index		1 (0–2)	4 (3–5)	0.000
**Outcome**	–			0.000
Survival, *N* (%)		947 (87.5)	492 (69.3)	
Death, *N* (%)		135 (12.5)	218 (30.7)	

AISI, aggregate index of systemic inflammation; ALP, Alkaline phosphatase; ALT, alanine transaminase; AST, aspartate transaminase; BG, blood glucose; Cr, creatinine; dNLR, derived neutrophil/lymphocyte ratio; ESR, erythrocyte sedimentation rate; Hb, hemoglobin; Hct, hematocrit; INR, international normalized ratio; MLR, monocyte/lymphocyte ratio; NLPR, neutrophil/lymphocyte*platelet ratio; NLR, neutrophil/lymphocyte ratio; PLR, platelet/lymphocyte ratio; PLT, platelet; PT, Prothrombin time; PTT, Partial thromboplastin time; SII, systemic inflammation index; SIR-I, systemic inflammation response index; WBC, white blood cell.

### Laboratory parameters

At the time of hospitalization, the laboratory tests performed are summarized in Table 1. Most of the tests performed in both groups were normal except for AST, LDH, ferritin, ESR, BS, urea, ALP, and Alb.

### Hematological tests

#### White blood cells and differential cells count

In elderly patients, WBCs, neutrophils, eosinophils, and monocytes counts were significantly higher than in non-elderly patients, while lymphocytes count significantly lower than non-elderly patients (*P* < 0.01 to *P* < 0.001) (Table 1).

#### Hemoglobin, hematocrit and platelet

In the elderly patients, the amount of Hb was significantly lower than in the non-elderly group (*P* < 0.01). There was no significant difference between Hct and Plt values between the two groups.

### Coagulation tests

#### Prothrombin time, partial thromboplastin time, and international normalized ratio

PT and INR levels were significantly higher in elderly than non-elderly patients (*P* < 0.001 for both), but there was no significant difference in PTT level.

### Biochemical tests

#### Blood glucose test

BG concentration in the elderly patients was significantly higher than in non-elderly patients (*P* < 0.001).

#### Liver enzymes

Alkaline phosphatase (ALP) level was significantly higher in the elderly patients than in the non-elderly (*P* < 0.05), while alanine transaminase (ALT) level was significantly higher in the non-elderly than elderly patients (*P* < 0.001). There was no significant difference in AST and LDH levels between the two groups.

#### Kidney tests

Urea and creatinine (Cr) levels were significantly higher in the elderly than non-elderly patients (for both, *P* < 0.001). In comparison, albumin level was significantly lower in the elderly than in the non-elderly patients (*P* < 0.01).

#### Electrolyte analysis

Sodium was significantly lower (*P* < 0.05) and potassium was significantly higher (P <0.001) in elderly compared to non-elderly patients.

#### Inflammatory markers

The erythrocyte sedimentation rate (ESR), ferritin, and D-Dimer levels were not significantly different between the two groups.

#### Systemic inflammatory index

NLR, MLR, dNLR, NLPR, SIR-I, SII, and AISI indices in the elderly patients were significantly higher than in the non-elderly patients (for all, *P* < 0.001).

### Clinical outcomes

Of the 1,082 non-elderly COVID-19 patients, 947 (87.5%) were discharged, and 135 died (12.5%). In the elderly group, 69.3% of patients were discharged, and 30.7% died. In terms of disease severity, in non-elderly patients, 12.5% of patients were very severe, 7.9% severe, and 79.6% moderate. But, in elderly patients, 25.6% of patients were very severe, 10.7% severe, and 63.7% moderate (Table 1).

### Laboratory parameters based on outcome

Table 2 shows the summary of laboratory findings in both non-elderly and elderly patients between deceased and surviving patients. In the non-elderly patients, the parameters that significantly were higher in the deceased than in the survivors were age, hospitalization stay, WBC count, neutrophil count, PT, PTT, INR, ALT, AST, LDH, ferritin, BG, urea, Cr, ALP, K, NLR, PLR, MLR, dNLR, NLPR, AISI, SIR-I, and SII, while lymphocytes count, eosinophil count, monocyte count, Hb, and Alb, were significantly lower.

**Table 2:** Demographic, hematological, and blood cell count-derived inflammation indexes of non-elderly and elderly COVID-19 in survivor and non-survivor patients.

**Variables**	**COVID-19**			
	**Non-elderly patients (*n* = 1,082)**		**Elderly patients (*n* = 710)**	
	**Survival**	**Death**	**Survival**	**Death**
Age	47.60 ± 11.75	53.63 ± 8.91^a^	75.58 ± 6.68^b^	77.89 ± 6.86^a, c^
**Sex**				
Male, *N* (%)	548 (86.2)	88 (13.8)	238 (67.6)	114 (32.4)
Female, *N* (%)	399 (89.5)	47 (10.5)	254 (70.9)	104 (29.1)
Hospitalization stay	6.98 ± 5.47	14.33 ± 13.89^a^	7.66 ± 5.18^b^	10.19 ± 9.28^a, c^
WBC (×10^9^/L)	7.50 (7.15–7.88)	7.72 (7.29–8.11)^a^	8.21 (7.88–8.62)^b^	8.33 (7.89–8.81)^c^
Neutrophil (×10^9^/L)	5.76 (5.01–6.40)	6.63 (6.06–7.11)^a^	6.53 (5.79–7.12)^b^	6.99 (6.45–7.58)^a, c^
Lymphocyte (×10^9^/L)	1.34 (0.85–1.91)	0.80 (0.50–1.19) ^a^	1.24 (0.84–1.83)	0.87 (0.61–1.28) ^a^
Eosinophil (×10^9^/L)	0.13 (0.07–0.17)	0.07 (0.06–0.14)^a^	0.15 (0.07–0.18)^b^	0.09 (0.07–0.17)^a, c^
Monocyte (×10^9^/L)	0.22 (0.14–0.32)	0.15 (0.09–0.23)^a^	0.24 (0.16–0.33)^b^	0.23 (0.12–0.33)^c^
Hb (mg/ml)	13.3 (12–14.6)	12.5 (10.8–14.1)^a^	12.9 (11.4–14.3)^b^	12.95 (11.4–14.50)
Hct (%)	39 (36–42.8)	39 (33–42.1)^a^	38.6 (34.6–42.2)^b^	39 (34.9–43.2)
PLT (×10^9^/L)	189 (147–249)	198 (148–261)	181 (141–249)	188 (131–252)
PT (s)	12.5 (12–13)	13 (12.5–14.4)^a^	12.5 (12–13.5)^b^	13.4 (12.5–14.5)^a^
PTT	30 (30–35)	33 (30–40)^a^	30 (30–37)	30 (30–38)
INR	1 (1–1.1)	1.1 (1–1.3)^a^	1 (1–1.10)^b^	1.1 (1–1.30)^a^
ALT (IU/L)	38 (25–57)	43 (30–69)^a^	30.5 (20–49)^b^	33 (23–55)^c^
AST (IU/L)	46 (32–65)	70 (50–95.5)^a^	45 (31–67)	58 (41–95)^a, c^
LDH (IU/L)	637 (480–786)	972 (708–1386)^a^	610 (470–775)	790 (595–1,075)^a, c^
Ferritin (μg/L)	600 (273–950)	890 (443–1,932)^a^	536 (231–965)	920 (434–1,474)^a^
ESR (mm/hr)	45 (31–62)	49 (34–69)	46 (30–62)	50 (34–65.5)
BG (mg/ml)	112 (98–145)	160 (119–241)^a^	129 (103–189)^b^	146 (114–219)^a^
Urea (mg/mL)	30 (23–40)	43 (34–65)^a^	48 (35–69)^b^	65.5 (47–94.5)^a, c^
Cr (mg/mL)	0.9 (0.8–1.1)	1.1 (0.9–1.3)^a^	1.1 (0.90–1.55)^b^	1.3 (1.05–2.2)^a, c^
D.Dimer (mg/L)	500 (160–1,011)	800 (250–2,500)	500 (250–900)	680 (250–2,000)
Albumin (g/mL)	3.6 (3.20–4)	3.30 (2.7–3.65)^a^	3.3 (3.15–3.85)	2.9 (2.5–3.3)^a, c^
ALP (IU/L)	170 (138–229)	200 (143–284)^a^	190 (149–246)^b^	207 (161–286)^a^
Na (mEq/L)	140 (137–142)	139 (136–142)	139 (136–141)^b^	139 (136–142)
K (mEq/L)	3.9 (3.6–4.2)	4.1 (3.8–4.5)^a^	4.1 (3.8–4.6)^b^	4.2 (3.8–4.9)^a, c^
NLR	4.33 (2.65–7.45)	8.60 (5.63–13.90)^a^	5.33 (3.17–8.5)^b^	8.5 (5.12–12.28)^a^
PLR	143 (88–243)	266 (138–438)^a^	149 (90–246)	216 (118–361)^a, c^
MLR	0.16 (0.09–0.26)	0.20 (0.11–0.34)^a^	0.18 (0.11–0.3)^b^	0.25 (0.12–0.37)^a^
SIR–I	0.90 (0.49–1.55)	1.39 (0.67–2.35)^a^	1.19 (0.70–2.02)^b^	1.70 (0.83–2.68)^a^
SII	821 (456–1,520)	1,708 (845–3,015)^a^	1,001 (527–1,703)^b^	1,493 (728–2,619)^a^
dNLR	3.34 (2.12–5.25)	6.14 (4–9)^a^	4 (2.57–6.14)^b^	5.66 (3.76–9)^a^
NLPR	0.02 (0.01–0.03)	0.03 (0.02–0.07)^a^	0.02 (0.01–0.04)^b^	0.04 (0.02–0.07)^a^
AISI	176 (81–320)	259 (105–569)^a^	226 (113–417)^b^	267 (139–577)^a^

Abbreviations similar to Table 1.

a P < 0.05, in both group survival vs. death patients;

b P < 0.05, non-elderly survival vs. elderly survival patients;

c P < 0.05, non-elderly death vs. elderly death patients.

In the deceased elderly patients, the parameters that significantly were high, including age, hospitalization stay, neutrophil count, PT, INR, AST, LDH, ferritin, BS, urea, Cr, ALP, K, NLR, PLR, MLR, dNLR, NLPR, AISI, SIR-I, and SII, while lymphocyte count, eosinophil count, and Alb levels were significantly decreased.

Hospitalization stay, WBC count, neutrophil count, ALT, AST, LDH, urea, Cr, K, and PLR were significantly higher in elderly death patients than in non-elderly deaths, while eosinophil count, monocyte count, and Alb levels were significantly lower.

### Receiver operating characteristics

**Figure 1:**
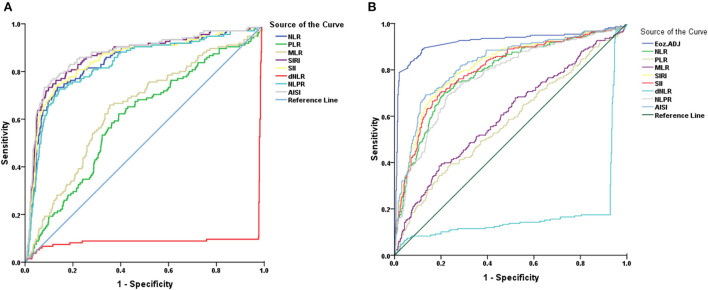
Receiver operating characteristics curve of **(A)** Non-elderly patients and **(B)** Elderly patients for NLR, PLR, MLR, dNLR, NLPR, AISI, SIR-I, and SII. AISI, aggregate index of systemic inflammation; dNLR, derived neutrophil/lymphocyte ratio; MLR, monocyte/lymphocyte ratio; NLPR, neutrophil/lymphocyte*platelet ratio; NLR, neutrophil/lymphocyte ratio; PLR, platelet/lymphocyte ratio; SII, systemic inflammation index; SIR-I, systemic inflammation response index.

ROC-based analysis to assess survival, the optimal cut-off values identified were as follows: WBC (non-elderly = 8.94 and elderly = 9.12), neutrophil count (non-elderly = 8.91 and elderly = 8.93), lymphocytes count (non-elderly = 1.23 and elderly = 0.64), NLR (non-elderly = 9.38 and elderly = 9.13), MLR (non-elderly = 0.26 and elderly = 0.36), PLR (non-elderly = 0.22 and elderly = 0.27), dNLR (non-elderly = 5.90 and elderly = 5.83), NLPR (non-elderly = 0.044 and elderly = 0.045), AISI (non-elderly = 492 and elderly = 518), SIR-I (non-elderly = 0.25 and elderly = 0.23), and SII (non-elderl y = 1,994 and elderly = 1,868) (Figure 1; Table 3).

**Table 3:** Receiver operating characteristics (ROC) curves and prognostic accuracy of blood cell count-derived inflammation indexes in non-elderly and elderly COVID-19.

**Variables**	**AUC**	**95% CI**	***p*-value**	**Cut-off**	**Sensitivity**	**Specificity (%)**
**WBC**						
Non-elderly	0.976	0.965–0.984	0.000	>8.94	93.3	96
Elderly	0.942	0.923–0.958	0.000	>9.12	85.8	94.3
All	0.969	0.960–0.977	0.000	>9.05	88.95	95.90
P1			0.013			
**Neutrophil**						
Non-elderly	0.981	0.971–0.988	0.000	>8.91	91.9	97.8
Elderly	0.943	0.924–0.959	0.000	>8.93	86.7	91.7
All	0.971	0.962–0.978	0.000	>8.79	89.8	94.3
P1			0.003			
**Lymphocyte**						
Non-elderly	0.578	0.548–0.608	0.003	<1.02	65.9	53.1
Elderly	0.551	0.513–0.588	0.036	<0.64	30.7	81.3
All	0.566	0.543–0.589	0.000	<0.91	50.4	61.2
P1			0.443			
**NLR**						
Non-elderly	0.839	0.815–0.860	0.000	>9.38	73.3	86.5
Elderly	0.785	0.753–0.814	0.000	>9.13	70.2	78.3
All	0.817	0.798–0.835	0.000	>9.21	71.39	83.1
P1			0.061			
**MLR**						
Non-elderly	0.644	0.615–0.673	0.000	>0.26	65.9	64.9
Elderly	0.603	0.566–0.639	0.000	>0.36	38.5	80.5
All	0.628	0.605–0.651	0.000	>0.26	59.4	62.4
P1			0.238			
**PLR**						
Non-elderly	0.603	0.573–0.632	0.000	>0.22	62.2	60.8
Elderly	0.575	0.537–0.612	0.002	>0.27	39.4	75.9
All	0.585	0.562–0.608	0.000	>0.23	52.6	63.1
P1			0.434			
**SIR-I**						
Non-elderly	0.864	0.842–0.884	0.000	>2.46	73.3	90.2
Elderly	0.813	0.783–0.841	0.000	>2.32	71.6	80.9
All	0.845	0.827–0.862	0.000	>2.32	72.8	85.7
P1			0.061			
**SII**						
Non-elderly	0.848	0.825–0.869	0.000	>1,994	74.1	87.2
Elderly	0.800	0.769–0.829	0.000	>1,868	70.2	80.4
All	0.826	0.807–0.843	0.000	>1,928	71.1	84.3
P1			0.091			
**dNLR**						
Non-elderly	0.900	0.880–0.917	0.000	<5.90	90.4	97.6
Elderly	0.821	0.791–0.848	0.000	<5.83	82.6	92.9
All	0.854	0.837–0.871	0.000	<5.83	85.5	95.9
P1			0.011			
**NLPR**						
Non-elderly	0.828	0.804–0.850	0.000	>0.044	72.6	85.7
Elderly	0.770	0.737–0.801	0.000	>0.045	69.3	76.9
All	0.807	0.788–0.825	0.000	>0.044	71.1	82.3
P1			0.048			
**AISI**						
Non-elderly	0.871	0.849–0.890	0.000	>492	76.3	89.1
Elderly	0.826	0.796–0.853	0.009	>518	69.3	86.5
All	0.852	0.835–0.868	0.000	>517	71.1	89
P1			0.090			

AISI, aggregate index of systemic inflammation; dNLR, derived neutrophil/lymphocyte ratio; MLR, monocyte/lymphocyte ratio; NLPR, neutrophil/lymphocyte*platelet ratio; NLR, neutrophil/lymphocyte ratio; PLR, platelet/lymphocyte ratio; SII, systemic inflammation index; SIR-I, systemic inflammation response index; WBC, white blood cell. P1, P-value of non-elderly vs. elderly patients.

In non-elderly group, AUD level was significant for WBC count (0.976), neutrophil count (0.981), lymphocyte count (0.578), NLR (0.839), MLR (0.644), PLR (0.603), dNLR (0.900), NLPR (0.828), AISI (0.871), SIR-I (0.864), and SII (0.848) (Figure 1A; Table 3). The WBC and neutrophil counts had a significantly higher AUC value than lymphocyte count [(z = 14.360, *P* < 0.001) and (z = 14.754, *P* < 0.001), respectively] in distinguishing dead from surviving patients. Regarding the systemic inflammatory index, it was also revealed that dNLR had a significantly higher AUC value than NLR (z = 2.058, *P* < 0.05), NLPR (z = 2.376, *P* < 0.05), PLR (z = 8.279, *P* < 0.001), and MLR (z = 7.259, *P* < 0.001) in distinguishing the dead from the surviving in non-elderly patients. In addition, SIR-I had a significantly higher AUC value than NLR (z = 8.967, *P* < 0.05), PLR (z = 15.717, *P* < 0.001), MLR (z = 14.697, *P* < 0.001), NLPR (z = 9.009, *P* < 0.001), and SII (z = 7.860, *P* < 0.001) in distinguishing the dead from the surviving in non-elderly patients.

On the other hand, in elderly patients, AUD levels were significant for WBC count (0.942), neutrophil count (0.943), lymphocyte count (0.551), NLR (0.785), MLR (0.603), PLR (0.575), dNLR (0.821), NLPR (0.770), AISI (0.826), SIR-I (0.813), and SII (0.800) (Figure 1B; Table 3). In the diagnosis of deceased elderly patients from the surviving, in relation to systemic inflammatory markers, it was identified that dNLR had a significantly higher AUC value than PLR (z = 7.790, *P* < 0.001), MLR (z = 7.012, *P* < 0.001), and AISI (z = 10.583, *P* < 0.001). In addition, SIR-I had a significantly higher AUC value than NLR (z = 11.775, *P* < 0.05), PLR (z = 16.690, *P* < 0.001), MLR (z = 16.061, *P* < 0.001), NLPR (z = 11.456, *P* < 0.001), and AISI (z = 7.335, *P* < 0.001) in distinguishing the dead from the surviving in old patients.

Interestingly, the comparison of AUD values for non-elderly and elderly COVID-19 patients revealed that the WBC count (*P* < 0.05), neutrophil count (*P* < 0.01), dNLR (*P* < 0.05), and NLPR values (*P* < 0.05) were significantly higher in non-elderly patients than in elderly patients (Table 3).

**Figure 2:**
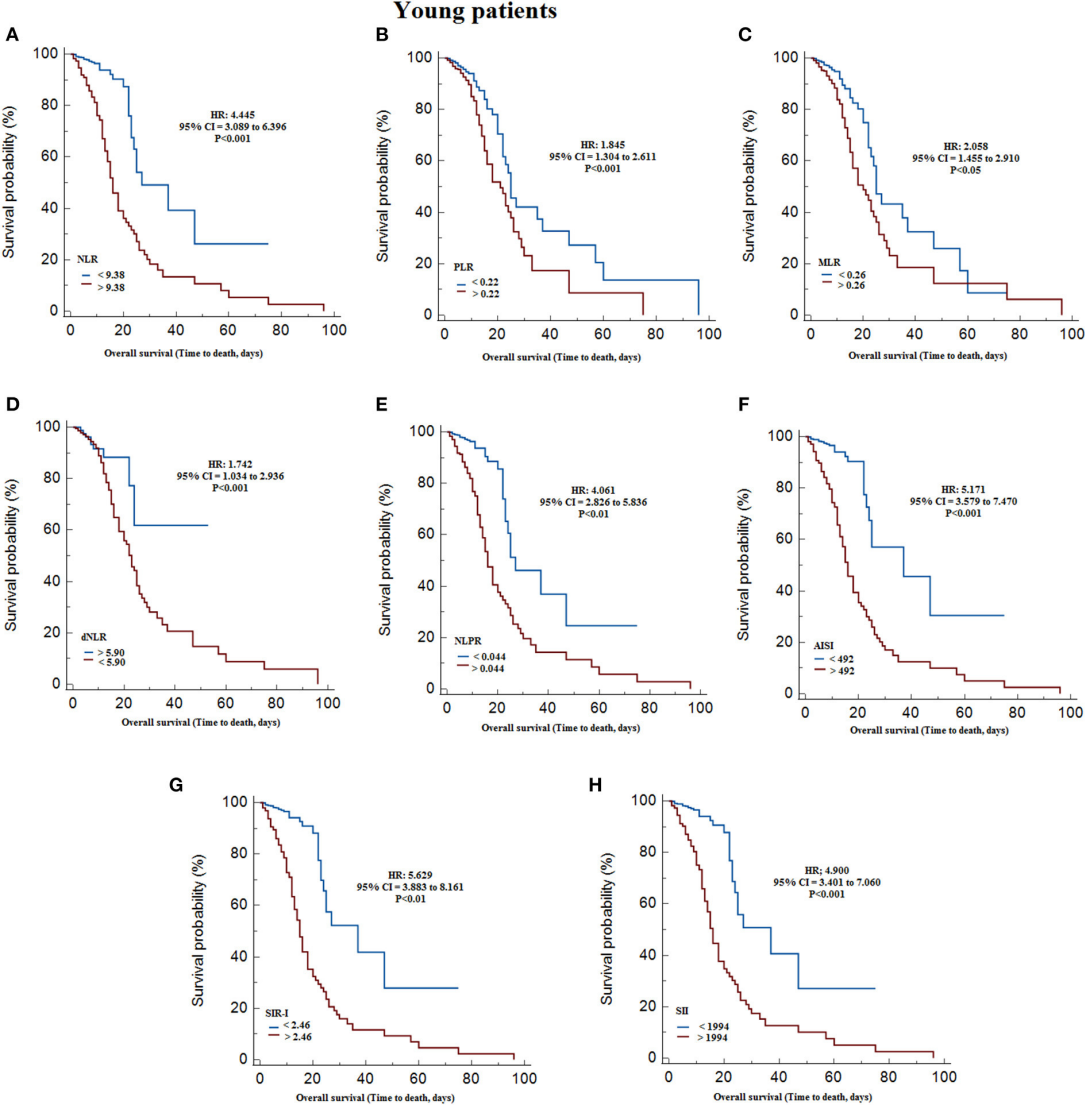
Kaplan–Meier survival curves during hospitalization of non-elderly COVID-19 patients with different cut-off values of the systemic inflammation indexes investigated. **(A)** NLR; **(B)** PLR; **(C)** MLR; **(D)** dNLR; **(E)** NLPR; **(F)** AISI; **(G)** SIR-I; **(H)** SII. AISI, aggregate index of systemic inflammation; dNLR, derived neutrophil/lymphocyte ratio; MLR, monocyte/lymphocyte ratio; NLPR, neutrophil/lymphocyte*platelet ratio; NLR, neutrophil/lymphocyte ratio; PLR, platelet/lymphocyte ratio; SII, systemic inflammation index; SIR-I, systemic inflammation response index; WBC, white blood cell.

**Figure 3:**
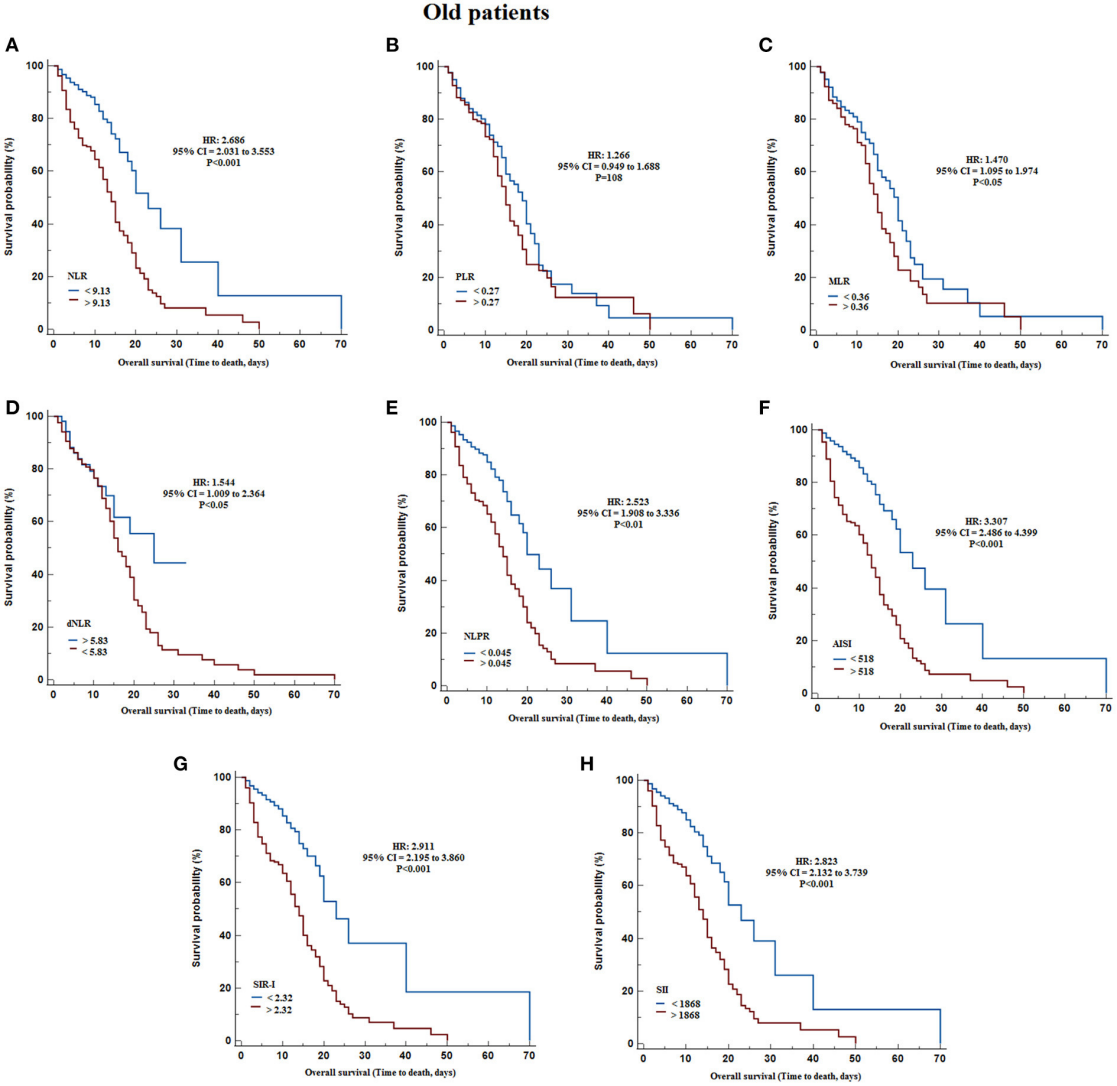
Kaplan–Meier survival curves during hospitalization of elderly COVID-19 patients with different cut-off values of the systemic inflammation indexes investigated. **(A)** NLR; **(B)** PLR; **(C)** MLR; **(D)** dNLR; **(E)** NLPR; **(F)** AISI; **(G)** SIR-I; **(H)** SII. AISI, aggregate index of systemic inflammation; dNLR, derived neutrophil/lymphocyte ratio; MLR, monocyte/lymphocyte ratio; NLPR, neutrophil/lymphocyte*platelet ratio; NLR, neutrophil/lymphocyte ratio; PLR, platelet/lymphocyte ratio; SII, systemic inflammation index; SIR-I, systemic inflammation response index.

According to Kaplan-Meier survival curves, after classifying non-elderly patients based on Youden cut-offs obtained with ROC curves, identified significantly lower survival with higher values of WBC count (HR = 16.381, 95% CI = 10.962 to 24.477, *P* < 0.001), neutrophil count (HR = 15.406, 95% CI = 10.377 to 22.870, *P* < 0.001), monocytes count (HR = 14.867, 95% CI = 9.978 to 22.150, *P* < 0.001), NLR (HR = 4.445, 95% CI = 3.089 to 6.396, *P* < 0.001), PLR (HR = 1.845, 95% CI = 1.304 to 2.611, *P* < 0.001), MLR (HR = 2.058, 95% CI = 1.455 to 2.910, *P* < 0.05), NLPR (HR = 4.061, 95% CI = 2.826 to 5.836, *P* < 0.001), AISI (HR = 5.171, 95% CI = 3.579 to 7.470, *P* < 0.001), SIR-I (HR = 5.629, 95% CI = 3.883 to 8.161, *P* < 0.001), and SII (HR = 4.900, 95% CI = 3.401 to 7.060, *P* < 0.001), and decreasing the lymphocytes count (HR = 1.620, 95% CI = 1.145 to 2.291, *P* < 0.001) and dNLR (HR = 1.742, 95% CI = 1.034 to 2.936, *P* < 0.05) (Table 4; Figure 2). On the other hand, in the elderly patients, the results revealed that survival was significantly reduced by increasing WBC count (HR = 7.350, 95% CI = 5.495 to 9.831, *P* < 0.001), neutrophil count (HR = 6.294, 95% CI = 4.708 to 8.414, *P* < 0.001), NLR (HR = 2.686, 95% CI = 2.031 to 3.553, *P* < 0.001), NLPR (HR = 2.523, 95% CI = 1.908 to 3.336, *P* < 0.001), AISI (HR = 3.307, 95% CI = 2.486 to 4.399, *P* < 0.001), SIR-I (HR = 2.911, 95% CI = 2.195 to 3.860, *P* < 0.001), SII (HR = 2.823, 95% CI = 2.132 to 3.739, *P* < 0.001), and decreasing the dNLR (HR = 1.544, 95% CI = 1.009 to 2.364, *P* < 0.05) (Table 4; Figure 3).

**Table 4:** Hazard ratios of the indexes under investigation obtained by Cox regression analysis in non-elderly and elderly COVID-19.

**Variables**	**HR**	**95% CI**	***p*-value**
**WBC**			
Non-elderly	16.381	10.962–24.477	0.000
Elderly	7.350	5.495–9.831	0.000
All	11.210	8.831–14.299	0.000
**Neutrophil**			
Non-elderly	15.406	10.377–22.870	0.000
Elderly	6.294	4.708–8.414	0.000
All	10.204	8.055–12.928	0.000
**Lymphocyte**			
Non-elderly	1.620	1.145–2.291	0.001
Elderly	1.195	0.879–1.626	0.254
All	1.260	1.016–1.562	0.035
**NLR**			
Non-elderly	4.445	3.089–6.396	0.000
Elderly	2.686	2.031–3.553	0.000
All	3.570	2.859–4.458	0.000
**MLR**			
Non-elderly	2.058	1.455–2.910	0.024
Elderly	1.470	1.095–1.974	0.013
All	1.502	1.212–1.860	0.000
**PLR**			
Non-elderly	1.845	1.304–2.611	0.000
Elderly	1.266	0.949–1.688	0.108
All	1.451	1.170–1.799	0.000
**SIR-I**			
Non-elderly	5.629	3.883–8.161	0.001
Elderly	2.911	2.195–3.860	0.000
All	4.088	3.268–5.115	0.000
**SII**			
Non-elderly	4.900	3.401–7.060	0.000
Elderly	2.823	2.132–3.739	0.000
All	3.682	2.945–4.604	0.000
**dNLR**			
Non-elderly	1.742	1.034–2.936	0.000
Elderly	1.544	1.009–2.364	0.045
All	0.618	0.446–0.856	0.004
**NLPR**			
Non-elderly	4.061	2.826–5.836	0.001
Elderly	2.523	1.908–3.336	0.001
All	3.327	2.667–4.151	0.000
**AISI**			
Non-elderly	5.171	3.579–7.470	0.000
Elderly	3.307	2.486–4.399	0.000
All	4.710	3.752–5.911	0.000

AISI, aggregate index of systemic inflammation; dNLR, derived neutrophil/lymphocyte ratio; MLR, monocyte/lymphocyte ratio; NLPR, neutrophil/lymphocyte*platelet ratio; NLR, neutrophil/lymphocyte ratio; PLR, platelet/lymphocyte ratio; SII, systemic inflammation index; SIR-I, systemic inflammation response index; WBC, white blood cell.

The multivariate Cox regression models in the non-elderly patients showed that WBC count (HR = 4.668, 95% CI = 1.624 to 13.413, *P* < 0.01), neutrophil count (HR = 6.395, 95% CI = 2.070 to 19.760, *P* < 0.01), dNLR (HR = 0.390, 95% CI = 0.182 to 0.835, *P* < 0.05), and SII (HR = 10.725, 95% CI = 1.076 to 106.826, *P* < 0.05) were significantly associated with survival. On the other hand, in elderly patients, it was found that WBC count (HR = 4.076, 95% CI = 2.176 to 7.637, *P* < 0.001) and neutrophil count (HR = 2.412, 95% CI = 1.252 to 4.647, *P* < 0.01) were significantly associated with survival.

## Discussion

The most important findings of the current study were:

1. Elderly patients had more severe laboratory results and systemic inflammatory indices (NLR, PLR, dNLR, SIR-I, SII, AISI, and NLPR) on admission compared to non-elderly patients.
2. ROC and Kaplan-Meier survival curves revealed that systemic inflammatory indicators in elderly and non-elderly patients were significantly associated with survival.
3. The multivariate Cox regression model showed that WBC count, neutrophil count, dNLR, and SII in non-elderly patients and WBC count and neutrophil count in elderly adults were significantly associated with survival.

COVID-19 disease has killed millions of people worldwide and has often affected health care systems in the worst-hit areas (16). In particular, the detrimental effects of the COVID-19 pandemic have been more severe in vulnerable individuals, including the elderly and patients with comorbidities (17). Identifying risk factors and using systemic inflammation indices in the diagnosis and progression of the COVID-19 disease can be effective in proper management and reducing mortality (18).

Preliminary laboratory findings showed that the results were more severe in elderly adults compared to non-elderly patients at admission. Based on the results, leukocytosis, neutrophilia, and lymphopenia were more common in elderly adults. Findings related to PT, INR, BUN, and Cr were also significantly higher in elderly patients, consistent with the previous lectures (10, 19, 20). As an indicator of disease severity, hospitalization in the ICU was found to be more common in elderly patients than in non-elderly patients, reflecting the rapid course and adverse outcome of COVID-19 disease in elderly patients (20). Most studies have shown that the male gender was an independent risk factor for death in COVID-19 patients (21). In the present study, although in both groups (elderly and non-elderly), the mortality rate was higher in men than women, there was no significant difference. Following hospitalization, another critical risk factor for death in COVID-19 patients was age (21). The present study results also showed that the mortality rate in elderly adults was significantly higher than in non-elderly patients (30.7 vs. 12.5%).

A variety of age-related physiological and immunological changes associated with comorbidities are influential factors in the elderly population that can lead to exacerbation of COVID-19 disease (22–24). The difference in mortality rates in non-elderly and elderly patients suggests that there may be several different risk factors that underlie this difference. With this in mind, we examined the ratios and some systemic inflammation indicators in predicting mortality in COVID-19 non-elderly and elderly patients. Many systemic inflammation indices have been considered in the diagnosis and progression of various diseases, especially inflammatory diseases (14).

The results of the present study identified that leukocytes count, neutrophil count, NLR, MLR, dNLR, NLPR, AISI, SIR-I, and SII were significantly higher in elderly adults than in non-elderly. In addition, neutrophilia and lymphopenia in both deceased non-elderly and elderly patients, along with increased levels of NLR, PLR, MLR, dNLR, NLPR, AISI, SIR-I, and SII, were evident compared to survivor individuals. It was also found that the neutrophils count, monocytes count, and PLR were higher in the deceased elderly patients than in the deceased non-elderly patients. As part of the immune system, neutrophils play a crucial role in defense against microbial and fungal infections (25). However, their role in defense against the virus is not fully understood. Human studies of COVID-19 have reported neutrophil infiltration into the lungs, although their importance in animal studies has not been observed (26).

Furthermore, lymphopenia was evident in both the deceased non-elderly and elderly patients, which is thought to be due to the effects of the virus on T cells infection by ACE2 receptors (27). T cell imbalance is crucial in diagnosing the severity of COVID19. Decreased levels of CD4 + and CD8 + T cells can increase some ratios, such as NLR (14).

In both elderly and non-elderly patients, based on AUC values and Kaplan – Meier survival curves, it was shown that survival was associated with leukocytes count, neutrophils count, lymphocytes count, monocytes count, NLR, PLR, SIR-I, SII, AISI, dNLR, and NLPR values. The neutrophils count, WBC count and dNLR values in non-elderly patients and neutrophils count, WBC count, and AISI values in elderly patients were the highest in predicting disease severity. Remarkably, multivariate Cox regression analysis revealed that WBC count, neutrophil count, dNLR, and SII remained significantly with survival in non-elderly patients, but in elderly patients, WBC count and neutrophil count. In fact, WBC count and neutrophil count in both non-elderly and elderly patients, were reliable predictors of mortality. The present study results showed that physiological and immunological differences between the elderly and the non-elderly were influential on the role of systemic inflammatory markers in predicting mortality in COVID-19 patients.

It should be noted that in previous studies, it has been reported that NLR is an inflammatory index directly related to age in healthy individuals and COVID-19 patients (28, 29). The difference in the studied population or the sample size is the possible reason for the difference between the results of the previous studies and the current study's findings. On the other hand, only NLR has been examined in previous studies, but the current study reported other inflammatory indicators alongside NLR. Interestingly, in the findings of Vafadar Moradi et al. (30) regarding the predictions of COVID-19 deaths, the WBC count was in line with our results.

This study had some limitations. First, the current retrospective study was performed using patients from a single institution. Second, this is a retrospective study, and the data are collected based on the electronic records of the hospital, the accuracy, and reliability of which varies between in hospitals. Third, although patients' tests were used at the time of admission to assess the systemic inflammation index, each patient could be at a different stage of the disease. Finally, the results of this study were reported over time, and different coronavirus variants may have influenced the results.

In conclusion, although systemic inflammation indexes are markers for diagnosing the severity of inflammatory diseases, they should be used with caution in COVID-19 patients. The results showed that the WBC count and neutrophil count were reliable markers for predicting non-elderly and elderly patients' mortality. It is interesting to note that the inflammatory indices differed in the diagnosis of mortality in non-elderly and elderly patients, so that dNLR index was very prominent in non-elderly people and AISI index in elderly people. Therefore, in COVID-19 patients, as shown by age-related clinical and laboratory differences, differences in predictors of mortality should also be considered.

## Data availability statement

The original contributions presented in the study are included in the article/supplementary material, further inquiries can be directed to the corresponding author/s.

## Ethics statement

The studies involving human participants were reviewed and approved by IR.ARUMS.REC.1399.615. Written informed consent for participation was not required for this study in accordance with the national legislation and the institutional requirements.

## Author contributions

MA, HG, and JM: literature search, proposal writing, data collection, analysis of data, interpretation of data, manuscript preparation, and review of manuscript. NJ, NF, and YM: proposal writing, data collection, analysis of data, and review of manuscript. All authors contributed to the article and approved the submitted version.

## Conflict of interest

The authors declare that the research was conducted in the absence of any commercial or financial relationships that could be construed as a potential conflict of interest.

## Publisher's note

All claims expressed in this article are solely those of the authors and do not necessarily represent those of their affiliated organizations, or those of the publisher, the editors and the reviewers. Any product that may be evaluated in this article, or claim that may be made by its manufacturer, is not guaranteed or endorsed by the publisher.
